# Detection of Human Papillomavirus Infection and p16 Immunohistochemistry Expression in Bladder Cancer with Squamous Differentiation

**DOI:** 10.1371/journal.pone.0093525

**Published:** 2014-03-27

**Authors:** Sung Han Kim, Jae Young Joung, Jinsoo Chung, Weon Seo Park, Kang Hyun Lee, Ho Kyung Seo

**Affiliations:** Center for Prostate Cancer, National Cancer Center, Goyang, Korea; Louisiana State University Health Sciences center, United States of America

## Abstract

**Objectives:**

To determine the potential association between HPV infection and the squamous cell component of urothelial carcinoma (UC) of the bladder and to validate p16 overexpression as a surrogate marker for HPV infection in these cancers among Koreans.

**Methods:**

We analyzed the presence of HPV infection using an HPV-DNA chip and the expression of p16 using immunohistochemistry in 47 subjects between July 2001 and March 2011. The study group (n = 35) included patients with squamous differentiation of UC of the bladder. The control group (n = 12) included patients with squamous metaplasia of the bladder.

**Results:**

Baseline characteristics of control and study groups were similar. HPV DNA detection rates were approximately 2-fold higher in the study than the control group (17.1% [6/35] versus 8.3% [1/12], respectively), but the difference was not statistically significant. P16 overexpression was detected in 16/35 (45.7%) study group and 1/12 (8.3%) control group samples (p = 0.034). Both HPV-positivity and p16 overexpression were present in 3/35 (8.8%) study group samples, but none of the control group (p = 0.295). In the study group, the percentage of HPV-positive cases who were non-smokers was 2-fold higher than the percentage of HPV-negative cases who were non-smokers (66.7% [4/6] versus 31.0% [9/29], respectively); however, statistical significance was not achieved due to the small sample size.

**Conclusions:**

HPV infection may be associated with UC of the bladder with squamous differentiation, especially in non-smokers. However, p16 expression does not appear to be a strong surrogate marker for evidence of HPV infection in this type of cancer.

## Introduction

Bladder cancer (BC) accounts for 3.2–4% of all cancers worldwide and approximately 90% of BC are urothelial carcinoma (UC). Although UC of the bladder often has focal squamous differentiation, it is differentiated from squamous cell carcinoma (SCC) of bladder, which contains solely keratin-forming carcinoma cells. BC composed of mixed urothelial and squamous phenotypes is called UC with squamous differentiation (UC/SCC) [1].

Among the known carcinogens of UC of the bladder, cigarette smoking is the most well-characterized risk factor. It is responsible for nearly half of BCs [2]. Other risk factors for BC include occupational exposure to a number of aromatic amines; infection-related carcinogenesis, such as schistosomal infection; and large doses of certain drugs, including cyclophosphamide and phenacetin [3]. However, a large number of BCs remain unexplained.

Recently, the role of viruses has received considerable attention as potential carcinogens, especially human papilloma virus (HPV). HPV is a small, circular, double-stranded DNA virus that infects stratified squamous epithelium and has been estimated linked to almost 10% of all cancers worldwide, especially a subset of SCCs of the vulva, penis, anus, and oropharynx [4]–[7]. Among the different HPV types, certain types, such as HPV-16 and HPV-18, have an established etiological role in development of anogenital cancers. Although HPV-16 and/or -18 genomic sequences were also identified in the urinary tract of female patients with recurrent and persistent urethritis and cystitis [8], [9], a number of studies have investigated the possibility that HPV infection is a risk factor contributing to UC of the bladder; however, their results have conflicted, so no definitive conclusions are possible [10]–[13].

A related issue involves the possibility that p16 protein is overexpressed in UC of the bladder. The expression of p16 is well known to be associated with high-risk HPV infection in cervical cancer and head and neck cancer and it has been suggested as a qualified surrogate marker for the identification of biologically active HPV infection [14]–[16]. However, despite many recent studies focusing on the SCC component of UC of the bladder, it remains unclear whether overexpression of p16 protein is associated with oncogenesis of UC of bladder [5], [10], [17]–[19].

Therefore, the aim of the present study was to determine the presence of HPV infection and to validate the possibility of p16 overexpression as a surrogate marker for HPV infection in UC/SCC of the bladder in Koreans smokers and non-smokers.

## Materials and Methods

Following approval of the study by our Institutional Review Board, 47 patients were selected from the archives of the hospital from July 2001 to March 2011 (IRB No. NCCNCS-12-643). All patients provided verbal informed consent. The written consent was not obtained because the IRB of National Cancer Center approved to exempt the written consent procedure. We examined paraffin-embedded tissue samples obtained during transurethral resection of the bladder or cystectomy. The study group consisted of 35 patients with mixed UC/SCC of the bladder and the control group consisted of 12 patients with squamous metaplasia of the bladder. Of the study group patients, 33 had evidence of mixed UC/SCC and two had extensive SCC with a history of UC. The UC of the bladder specimens were classified according to the World Health Organization (WHO) and International Society of Urological pathology (ISUP) grading criteria and staged according to the TNM 2010 version. None of the patients had known or suspected histories of Schistosoma heamatobium infection, uterine cervix SCC, or head and neck SCC.

A representative SCC component of each paraffin block sample was selected based on the presence of adequate quality and quantity of extracted DNA. DNA was extracted using Qiagen BioRobot M48 workstation (Qiagen, CA) and the quality and quantity of DNA was measured using Nanodrop ND 1000 (NanoDrop Technologies, Wilmington, DE). The presence of HPV infection was analyzed using an HPV-DNA chip. HPV genotyping was performed as per the manufacturer's protocol using a polymerase chain reaction (PCR)-based DNA microarray system (Greencross, Gyeonggi, Korea), consisting of multiple probes of the HPV L1 sequence [20]. These probes included specific probes for high-risk HPV types (HPV-16, 18, 31, 33, 35, 39, 45, 51, 52, 53, 56, 58, 59, 66, and 68) and low-risk HPV types (HPV-6, 11, 34, 40, 42, 43, 44, 54, and 70). Consensus PCR products were hybridized to the probes on the chip, scanned (Scanner GX, PerkinElmer, MA), and then analyzed. PCR amplification was performed using 10 μL purified DNA.

**Figure 1:**
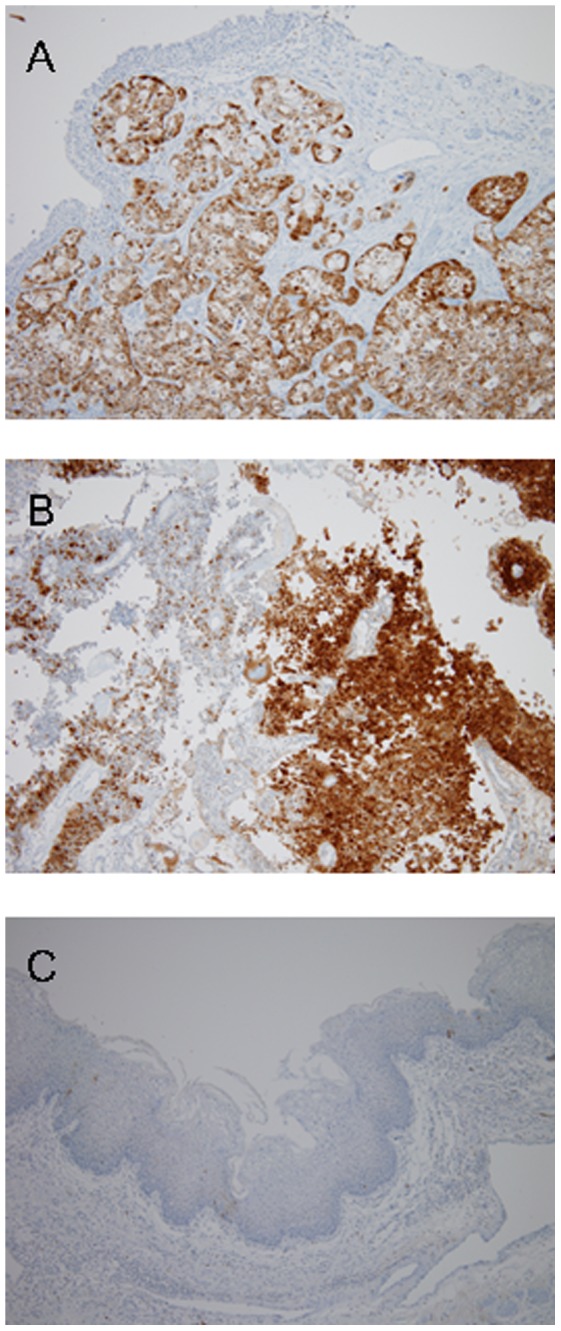
Histological findings and expression patterns of p16. A: The squamous cell carcinoma components are located beneath the urothelial lining. The tumor cells are strongly positive for p16 (x100). B: This case is a mixed papillary urothelial carcinoma (left upper) and squamous cell carcinoma. The squamous cell carcinoma component is strong positive for p16. C: Squamous metaplasia shows no expression of p16.

Immunohistochemistry (IHC) staining of p16 protein was performed using specific E6H4 antibodies (DAKO, Carpinteria, CA). IHC p16 expression was scored using a semiquantitative composite scoring system as follows: (1) staining intensity, defined as 0 for negative, 1+ for weak, 2+ for moderate, and 3+ for strong; (2) positive area, defined as the (10 X) fraction of stained tumor cells in the entire tumor; and (3) expression score, defined as the staining intensity multiplied by the positive area. The highest possible score was 30. Overexpression of p16 was defined as a score >20 (Figure 1).

The relationships between the presence of HPV infection and clinicopathological parameters were assessed using Fisher's exact tests and Mann-Whitney tests. All statistical analyses were performed using STATA (release 9.2, STATA Inc, Tex, USA). Results were considered statistically significant if the two-sided p value was <0.05.

## Results

Clinicopathological characteristics of the 35 UC/SCC study group patients and 12 squamous metaplasia control group patients are shown in Table 1. No demographic differences were observed between the study and control groups, including age (71.2±7.7 vs. 66.5±10.1 years, p = 0.246), male to female ratio (82.9% vs. 66.7%, p = 0.251), and smoking history (62.9% vs. 41.7%, p = 0.311). In the study group, 4 (12.1%) patients had low-grade carcinoma and 29 (87.9%) had high-grade. For the remaining 2 patients, the available samples (obtained by transurethral resection of the bladder) contained only an SCC component, thereby prohibiting the assignment of a grade.

**Table 1:** Demographic and histopathological characteristics of the study group and control group.

	Study group	Control group	*p*-value
No. of patients	35	12	
Age (years)	71.2±7.7	66.5±10.1	0.247
Sex			
Male	29 (82.9%)	8 (66.7%)	0.251
Female	6 (17.1%)	4 (33.3%)	
Stage			
NMIBC	8 (22.9%)	NA	NA
MIBC	17 (48.6%)	NA	NA
MBC	10 (28.6%)	NA	NA
Grade			
Low	4 (12.1%)	NA	NA
High	29 (87.9%)	NA	NA
NA	2	NA	NA
Smoking history			0.311
Smoker or Ex- smoker	22 (62.9%)	5 (41.7%)	
Non-smoker	13 (37.1%)	7 (58.3%)	

NMIBC: non-muscle invasive bladder cancer.

MIBC: muscle invasive bladder cancer.

MBC: metastatic bladder cancer.

NA: not applicable.

HPV DNA was detected in 6 of the 35 (17.1%) study group samples, and 1 of the 12 (8.3%) control group samples (Table 2). All HPVs were type 18, except for 1 study group sample, which was type 35. Overexpression of p16 was detected in 16 (45.7%) study group samples and 1 (8.3%) control group sample (Table 2). Both HPV positivity and p16 overexpression were present in only 3 (8.8%) study group samples and no control group sample.

**Table 2:** Detection of human papillomavirus and overexpression of p16 in the study group and control group.

	Study group	Control group	*p*-value
No. of patients	35	12	
HPV+	6 (17.1%)	1 (8.3%)	0.659
P16+	16 (45.7%)	1 (8.3%)	0.034
HPV+/P16+	3 (8.6%)	0 (0.0%)	0.295

HPV: human papillomavirus virus.

HPV+: detection of HPV.

P16+: overexpression of p16.

For the study group, there were no differences in age, sex, stage, or grade between HPV-negative and HPV-positive cases. Of the 29 HPV-negative cases, 20 (69.0%) were smokers or ex-smokers, whereas of the 6 HPV-positive cases, only 2 (33.3%) were smokers or ex-smokers. For smokers or ex-smokers, the percentage with HPV-negative BC was approximately 2-fold higher than the percentage with HPV-positive BC. By contrast, the BC samples of non-smokers demonstrated an approximately 2-fold higher percentage of HPV-positivity than HPV-negativity (Table 3).

**Table 3:** Demographic and histopathological characteristics according to detection of human papillomavirus in the study group.

	Study group (N = 35)		*p*-value
	HPV- (n = 29)	HPV+ (n = 6)	
Age (years)	71.4±7.9	70.0±6.5	0.718
Sex			0.973
Male	24 (82.8%)	5 (93.3%)	
Female	5 (17.2%)	1 (16.7%)	
Stage			0.220
NMIBC	6 (17.0%)	2 (5.8%)	
MIBC	16 (45.7%)	1 (2.9%)	
MBC	7 (20.0%)	3 (8.6%)	
Grade			0.571
Low	3 (11.1%)	1 (16.7%)	
High	24 (88.9%)	5 (83.3%)	
NA	2	0	
Smoking history			0.286
Positive+	20 (69.0%)	2 (33.3%)	
Negative	9 (31.0%)	4 (66.7%)	

NMIBC: non-muscle invasive bladder cancer.

MIBC: muscle invasive bladder cancer.

MBC: metastatic bladder cancer.

NA: not applicable.

+ , Positive included with all of the present and ex-smokers.

## Discussion

In addition to cigarette smoking, occupational exposure to aromatic amines, and use of specific drugs, such as cyclophosphamide and phenacetin, as known risk factors of BC, HPV infection has also been suggested as a potential causative agent of UC of the bladder [21], [22]. The prevalence of HPV in UC of the bladder reported in previous studies has varied from 0% to 81.3% [23]–[25]. The disparity is probably due to sampling problems, contamination, sensitivity of the detection systems, and geographic variation [26].

Youshya et al. [21]. reported that 60% of patients were positive for HPV L1 capsid protein expression by immunostaining, even though PCR using consensus GP5+/6+ primers failed to detect HPV DNA. The suggested reason for the disparity of HPV detection rates was the antibodies that were utilized and the sensitivity of analysis for detection of DNAs [27]. In this study, we utilized the HPV DNA chip microarray system. The HPV oligonucleotide microarray system is a newly developed biotechnology that can be applied in clinical practice for detection and genotyping of HPV. The HPV-DNA chip microarray has shown higher sensitivity for detection of HPV DNA than PCR alone in tissue block [28], [29].

The HPV has an affinity for squamous cells and its association with human SCC of the uterine cervix, head and neck, and anus has been well established [7]. In the current study, the detection rate of HPV DNA was 2-fold higher in the study group with mixed UC/SCC of the bladder (17.5%) than the control group with squamous dysplasia (8.3%). However, we could not confirm a statistically significant increase in risk of HPV infection in the study group, likely because of the relatively small sample size (Table 2). Our findings are consistent with other studies in which HPV DNA was detected in both UC of the bladder and SCC of the bladder [30].

Although HPV infection has been associated with BC in many studies, it is important to realize that such an association is not equivalent to causation. None of the previous studies have proven the existence of a direct link between HPV infection and carcinogenesis in BC, although the possibility of a causal role has been suggested because of the similarity to cervical and head and neck cancers, for which a causal relationship has been established [19] [31], [32]. To address this issue, we tried to validate p16 overexpression as a surrogate marker for active HPV infection in BC; however, we were unable to detect a strong association between p16 overexpression and HPV infection in UC/SCC of the bladder(Table 2).

HPV encodes two oncoproteins: E6 and E7 [33]. E6 protein binds to wild type (wt) p53, thus triggering ubiquitin-mediated degradation of p53 or directly inactivating wt-p53 by complex formation [34], [35]. E7 binds to pRb and releases the E2F transcription factor, which subsequently facilitates cell proliferation. P16 is a cyclin-dependent kinase (CDK) inhibitor that blocks CDK4-mediated phosphorylation of pRb and subsequent G1 to S progression. HPV-infected cells overexpress p16 in an attempt to compensate for the E7-induced loss of pRb. Because of these negative feedback mechanisms between pRb and p16, overexpression of p16 is now often used as a surrogate marker of HPV oncogene expression. In this study, p16 was overexpressed in 45.7% of UC/SCC samples; however, both HPV-positivity and p16 overexpression was present in only 8.6%. This finding thereby shows that p16 expression might not be a strong surrogate marker for evidence of HPV infection in UC/SCC, which is consistent with the results of Alexander et al. [10].

Additional findings of interest in this study were the types of HPV and the higher percentage of non-smokers in the HPV-positive group than in the HPV-negative group. Almost all HPV-positive samples in the study group were type 18. The sole exception was a sample with HPV type 35, which has been considered a variant of HPV 16 and which has not been previously isolated from the bladder [36]. As it is well known that the HPV types 16 and 18 are categorized as high risky HPV for a leading cause of cancer among various HPV types [7], [16], [37], [38], our result with HPV types 18 and 35 might be supported our hypothesis of HPV infection in association with the carcinogenesis of bladder cancer. Although the increased likelihood of HPV-positivity in non-smokers did not reach statistical significance because of the small sample size, this finding suggests that HPV infection might further be associated with the development of mixed UC/SCC of urinary bladder, especially in non-smokers because of its twice greater the percentage of HPV positivity than that of smokers (p = 0.286, Table 3).

This study had some limitations. A retrospective study with a relatively small number of samples limited its statistical power and potential bias. The serology to HPV suggested by Badawi could not be performed because of the study design, even though the antibody response (immunoglobulin [Ig]G) to L1 capsids could be used as a marker of cumulative HPV exposures [9]. The control group had squamous metaplasia, whereas a control group with normal urothelium may have been more appropriate to show a strong association between HPV infection and carcinogenesis of UC/SCC. However, this study is significant in determination of the relationship of HPV infection with prevalence of BC as one of causative factors in carcinogenesis of bladder and this is the first paper describing the clinical importance of the possible association between HPV infection and bladder cancer in non-smokers.

## Conclusion

Based on the findings of this study, HPV infection appears to be associated with the development of mixed UC/SCC of the urinary bladder, especially in non-smokers among Koreans. However, the role of HPV infection in the development of this type of cancer may not be as significant as for SCC of the uterine cervix or the head and neck. Additionally, p16 expression does not appear to be a strong surrogate marker for evidence of HPV infection in UC of the bladder with squamous differentiation.
